# Cytokine correlations in childhood-onset rheumatic diseases with pulmonary involvement

**DOI:** 10.3389/fped.2024.1441890

**Published:** 2024-11-12

**Authors:** Hua Huang, Fei Ding, Chenxi Liu, Shengfang Bao, Yingying Jin, Yanliang Jin, Yixiao Bao

**Affiliations:** ^1^ Department of Rheumatology and Immunology, Shanghai Children's Medical Center, Shanghai Jiao Tong University School of Medicine, Shanghai, China; ^2^ Shanghai Tonxin Clinic, Shanghai, China

**Keywords:** rheumatic diseases, connective tissue disease, cytokines, lung, children

## Abstract

**Background:**

There was less research about cytokines in lung involvement of childhood-onset rheumatic diseases (RD) patients.

**Objective:**

In this investigation, blood levels of 12 cytokines were tested in order to explore their associations with lung involvement in newly diagnosed childhood-onset RD patients.

**Methods:**

High-resolution computed tomography (HRCT) and pulmonary function tests (PFTs) were performed on 53 newly diagnosed RD patients without any treatment in our department between July 2021 and December 2023. Patients were separated into groups based on whether their lungs were involved or not according to abnormalities found on HRCT or PFTs. We examined the serum cytokines with 41 healthy, age and gender-matched controls.

**Results:**

The majority of serum cytokine levels were statistically different between the RD group with lung involvement and the control group. The RD group with lung involvement had considerably higher serum IL-6 and IFN-γ levels than the RD group without lung involvement or control group. The median serum IL-6 levels were 19 pg/ml [interquartile range (IQR) 6.9, 27.6], 4.4 pg/ml (IQR 2.0, 10.5), and 2.0 pg/ml (IQR 2.0, 2.0) in RD group with lung involvement, RD group without lung involvement, and control group, respectively. Similarly, the median serum IFN-γ levels were 6.0 pg/ml (IQR 5.0, 15), 5.0 pg/ml (IQR 5.0, 5.0), and 5.0 pg/ml (IQR 5.0, 5.0) in RD group with lung involvement, RD group without lung involvement, and control group, respectively. The receiver operating characteristic (ROC) curve study showed that IL-6 and IFN-*γ* had area under the curve (AUC) values of 0.782 and 0.742, respectively, indicating their significant diagnostic potential for lung involvement in RD patients.

**Conclusion:**

Our findings imply that IL-6 and IFN-γ may be associated with the development of lung illnesses and may be involved in the pathophysiology of RD. Thus, in individuals with RD, elevated levels of IFN-*γ* over 5.42 pg/ml or IL-6 above 6.25 pg/ml may warrant suspicion of lung involvement.

## 1 Introduction

Pediatric rheumatic diseases (RD) encompasses a variety of chronic autoimmune, autoinflammatory, and noninflammatory musculoskeletal conditions (1). No matter what the underlying conditions are, almost all rheumatic diseases could cause pulmonary symptoms, either as a result of the disease itself or as a side effect of pharmacological therapies (2). Pulmonary manifestations maybe present with RD or precede the diseases.

Interstitial lung disease (ILD) is a common manifestation of lung involvement in RD patients, particularly in illnesses such as systemic sclerosis (SSc), rheumatoid arthritis (RA), dermatomyositis (DM), systemic lupus erythematosis (SLE), Sjogren's syndrome (SS), and undifferentiated rheumatoid disorders (3). Up to half childhood-onset SSc patients could have lung involvement, such as ILD and pulmonary arterial hypertension (PAH) (4). Patients with systemic juvenile idiopathic arthritis (SJIA), particularly those with refractory disease histories and exposure to several medications, may develop potentially fatal pulmonary consequences such as pulmonary alveolar proteinosis (PAP), PAH, and ILD (5).

Although pulmonary problems are usually less common in children than in adults, they can lead to functional impairment and increased mortality (6). Clinical presentations might vary from being asymptomatic to having respiratory symptoms and exacerbations, which are more typical clinical presentations (7). The approaches employed for illness identification are relevant to the detection of pulmonary involvement. Determining dependable methods would support early intervention, especially in individuals who are asymptomatic.

The most reliable method for diagnosing RD-ILD is High-resolution computed tomography (HRCT). It is believed that the subclinical radiographic prevalence rate ranges from 33 to 57%, while the total incidence of ILD in connective tissue disease (CTD) is 15% (8). However, in young children, CT examinations pose problems with radiation exposure and anesthesia (9).

Pulmonary function tests (PFTs) is helpful in monitoring the degree and course of respiratory involvement and in identifying subclinical disease (10). But PFT need the cooperation of the patients.

For children, it would be ideal to identify noninvasive, trustworthy biomarkers that may be used to forecast the onset, prognosis, and reaction to therapy of disorders. According to some research, cytokines are crucial in the lung tissue damage, inflammation, and fibrosis that adult CTD-ILD patients experience (8, 11–14). However there was little research about cytokines in the pathogenesis of lung involvement in children with RD (5, 15, 16).

In order to investigate the relationship between alterations in cytokine levels and lung involvement in childhood patients with newly diagnosed RD, we measured the blood levels of 12 cytokines in both the RD children and the healthy controls in this investigation. We try to find some cytokines as potential diagnostics serum biomarkers.

## 2 Methods

### 2.1 Study population

This cross-sectional study was carried out between July 2021 and December 2023 at the Department of Rheumatology and Immunology, Shanghai Children's Medical Center, School of medicine, Shanghai Jiao Tong University.

Every participant provided written informed permission. The investigation was approved by the Research Ethics Committee of Shanghai Children's Medical Center, School of Medicine, Shanghai Jiao Tong University (SCMCIRB-W2021040).

Ninty-four participants were enrolled in this study. Fifty-three patients (eighteen patients with juvenile idiopathic arthritis [JIA], eleven with juvenile systemic lupus erythematosus [JSLE], nine with juvenile dermatomyositis [JDM], six with vasculitis (three with IgA vasculitis, one with Takayasu arteritis, one with Behçet's disease, one with ANCA-associated vasculitis), five with Sjogren's syndrome [SS], four with undifferentiated connective tissue disease [UCTD]) were newly diagnosed with RD (mainly including JIA, CTD, vasculitis) and were not on any treatment. All patients fulfilled the internationally accepted criteria of JIA, JSLE, JDM, SS, vasculities and UCTD and underwent evaluation using PFTs and HRCT. Based on the PFTs or HRCT abnormalities, the RD group was divided into RD with lung involvement and RD without lung involvement.

Forty-one healthy controls were recruited from individuals receiving examinations at the developmental and behavioral clinic of Shanghai Children's Medical Center. The controls matched the study group in age and sex distribution. Chronic lung, cardiac, or rheumatic illnesses were not seen in these controls. A medical checkup turned up nothing unusual.

Exclusion criteria encompassed individuals with respiratory symptoms and signs, active systemic diseases like malignancies, infections, metabolic diseases, and a history of chronic respiratory or cardiovascular conditions.

### 2.2 Laboratory analysis

Upon admission, we examined the serum concentrations of TNF-α, IFN-α, IFN-*γ*, and IL-1β, IL-2, IL-4, IL-5, IL-6, IL-8, IL-10, IL-12p70, IL-17. All patients and controls had venous blood samples (3 ml) drawn into standard blood collection tubes, which were then left to solidify for 30 min or more at room temperature. The samples were centrifuged for ten minutes at 1,000 rpm, and then they were kept at −20°C until analysis. Using a commercially available fluorescence flow cytometry assay kit (MAGPIX®, USA), cytokine concentrations were determined in accordance with the manufacturer's instructions.

### 2.3 Disease assessments

All patients underwent PFTs and pulmonary HRCT. PFT measurements were conducted in accordance with standards set by American Thoracic Society/European Respiratory Society (17).

At least three repetitions of each measurement were made, and the highest measurement that was found to be acceptable was compared to the expected normal values.

The definitions of pulmonary dysfunction were as follows: small airway disease (FEF75 or FEF75, and FEF50 < 65% of that predicted), isolated impairment of diffusion capacity (DLco < 80% of that predicted), obstructive dysfunction (FEV1/FVC < 80% of that predicted, and FEV1 < 80% of that predicted), and restrictive dysfunction (VC < 80% of that predicted).

HRCT was evaluated by two trained radiologists. The primary findings on HRCT were considered to be ground-glass opacity, consolidation, septal thickening, honeycombing, crazy paving, nodules, pleural and pericardial effusions, progressive volume loss, and pulmonary embolism (18).

### 2.4 Statistical analysis

Numerical variables were presented as mean and standard deviation (SD) or median and interquartile range [IQR; (Q1, Q3)]. Categorical variables were presented as the number of cases (percentage). Student's *t*-test for normally distributed values, Mann–Whitney *U*-test for nonnormally distributed data, Chi-square test or Fisher's exact test for categorical variables were uesed to compare patients and controls. To assess the relationships between cytokines and clinical indicators, Spearman correlation analysis was employed. To evaluate cytokines’ sensitivity and specificity in predicting lung involvement in patients with RD, receiver operating characteristic (ROC) curve analysis was performed.

IBM SPSS 26.0 software (IBM, Armonk, New York, USA) was used for all statistical analyses. *P* < 0.05 (two-sided) was used as the statistical significance threshold.

## 3 Results

### 3.1 Demographic and clinical characteristics

The demographic and clinical characteristics are listed in Table 1.

**Table 1:** Demographic and clinical characteristics.

Variables	RD group with lung involvement (*n* = 22)	RD group without lung involvement (*n* = 31)	*P*_ab_-value	RD (*n* = 53)	Controls (*n* = 41)	*P*_cd_-value
Age (years)	10.7 ± 4.0	10.1 ± 3.0	0.2	10.3 ± 3.4	9.8 ± 1.4	0.0734
Sex (female/male)	16 (73%)/6 (27%)	15 (48%)/16 (52%)	0.096	31 (58%)/22 (42%)	23 (56%)/18 (44%)	0.8363
Height (cm)	139.1 ± 11.9	136.0 ± 17.2	0.5763	137.2 ± 15.3	131.5 ± 12.8	0.0838
Weight (kg)	36.3 ± 11.6	36.5 ± 12.3	0.8606	36.5 ± 11.9	32.9 ± 7.3	0.1294
BMI	16.2 ± 2.2	17.6 ± 2.6	0.1161	17.1 ± 2.5	17.5 ± 2.5	0.4561
Duration (days)	30 [14, 112]	60 [30, 210]	0.2	60 [21, 180]	NS	
Illness			0.001*		NS	
SLE	10 (45.5%)	1 (3.2%)		11 (20.8%)		
JIA	4 (18.2%)	14 (45.2%)		18 (34.0%)		
JDM	4 (18.2%)	5 (16.1%)		9 (17.0%)		
Others	4 (18.2%)	11 (35.5%)		15 (28.3%)		

BMI, body mass index; SLE, systemic lupus erythematosus; JIA, juvenile idiopathic arthritis; JDM, juvenile dermatomyositis; RD, rheumatic disease.

* *P* < 0.05 was considered statistically significant.

Thirty-one female and twenty-two male RD patients made up the participants with a mean age of 10.3 ± 3.4. Data from 41 healthy control participants (including 23 females and 18 males) with the mean age of 9.8 ± 1.4 were compared with theirs. Age, weight, height, BMI and sex ratio did not significantly differ between the patients and control groups.

Based on subgroup analysis, the RD group included 18 patients with JIA, eleven with SLE, nine with JDM, six with vasculitis (three with IgA vasculitis, one with Takayasu arteritis, one with Behçet's disease, one with ANCA-associated vasculitis), five with SS, and four wtih UCTD. The median span from the start of the illness to the diagnosis was 60 days [21, 180].

According to the abnormalities of HRCT or PFTs, the RD group was divided into with or without lung involvement. There were 22 patients (mean age 10.7 ± 4.0 years) in the RD group with lung involvement, 16 of whom were female and 6 of whom were male. Thirty days [14, 112] was the median disease duration. There were 31 patients (mean age 10.1 ± 3.0 years) in the RD group without lung involvement, 15 of whom were female and 16 of whom were male. The median disease duration was 60 days [30, 210].

Notably, all RD patients with lung involvement were asymptomatic.

### 3.2 Pulmonary function tests and HRCT findings in RD patients

HRCT and PFTs were conducted for all patients with RD. Among the 53 patients, nine (17.0%) exhibited abnormal HRCT, and 15 (28.3%) had abnormal PFTs. Overall, 22 patients (41.5%) demonstrated lung involvement. Of the nine patients with abnormal HRCT, 5 (55.6%) were female, while among the fifteen patients with abnormal PFTs, 13 (86.7%) were female. Two patients (3.8%) exhibited HRCT and PFT abnormalities, and both were females.

The three types of radiological abnormalities were pleural thickening (*n* = 1), linear opacities (*n* = 1), and ground-glass opacities (*n* = 7).

PFTs abnormalities included restrictive dysfunction (*n* = 4), reduction of DLCO (*n* = 9) and small airway disease (*n* = 2).

### 3.3 Concentrations of 12 cytokines in serum

The RD group with lung involvement's serum levels of IL-2, IL-4, IL-5 as well as those of IL-6, IL-8, IL-10, IL-17, IFN-*γ* were statistically different from the control group's (*P* < 0.05) (Table 2).

**Table 2:** Differences of 12 cytokines between groups.

Cytokines	RD group with lung involvement (pg/ml) (*n* = 22)	RD group without lung involvement (pg/ml) (*n* = 31)	Controls (pg/ml) (*n* = 41)	*P*_ab_-value	*P*_ac_-value	*P*_bc_-value
IL-1β	3.0 [2.4, 3.3]	3.0 [2.4, 3.1]	3.1 [3.0, 3.7]	0.813	0.924	0.062
IL-2	4.0 [2.4, 4.3]	4.0 [2.4, 4.0]	4.0 [4.0, 4.0]	0.852	0.036*	0.705
IL-4	2.7 [2.4, 5.5]	3.0 [2.5, 16.9]	16.9 [10.3,33.2]	0.3	0.000*	0.000*
IL-5	3.0 [2.4, 4.0]	3.0 [2.4, 4.9]	6.5 [3.9, 19.5]	0.7	0.000*	0.000*
IL-6	19.0 [6.9, 27.6]	4.4 [2.0, 10.5]	2.0 [2.0, 2.0]	0.010*	0.000*	0.000*
IL-8	23.1 [7.9, 56.4]	22.0 [3.0, 39.5]	3.5 [3.3, 4.8]	0.5	0.000*	0.000*
IL-10	4.6 [2.7, 8.7]	4.5 [2.4, 5.6]	3.6 [3.1, 4.9]	0.5	0.021*	0.832
IL-12p70	2.4 [2.4, 4.6]	4.0 [2.4,8.8]	4.0 [4.0, 4.0]	0.3	0.638	0.169
IL-17	3.7 [2.4, 6.2]	5.0 [2.8, 5.0]	5.0 [5.0, 5.0]	0.782	0.007*	0.937
TNF-α	4.0 [2.4, 4.7]	4.0 [2.4, 4.0]	4.0 [4.0, 4.0]	0.11	0.647	0.384
IFN-α	5.0 [3.1, 16.5]	5.0 [2.4, 5.0]	5.0 [5.0, 11.5]	0.3	0.853	0.037*
IFN-*γ*	6.0 [5.0, 15]	5.0 [5.0, 5.0]	5.0 [5.0, 5.0]	0.009*	0.000*	0.229

IL, interleukin; TNF, tumor necrosis factor; IFN, interferon; RD, rheumatic disease.

* *P* < 0.05 was considered statistically significant.

Furthermore, the RD group with lung involvement had substantially higher levels of serum IL-6 and IFN-γ than the RD group without lung involvement (*P* < 0.05) (Table 2).

Specifically, 19.0 pg/ml (IQR 6.9, 27.6) in the RD group with lung involvement, 4.4 pg/ml (IQR 2.0, 10.5) in the RD group without lung involvement and 2.0 pg/ml (IQR 2.0, 2.0) in the control group were the median serum IL-6 levels. The RD group with lung involvement had IL-6 levels that were considerably higher than the control group and RD group without lung involvement (*P* < 0.05).

6.0 pg/ml (IQR 5.0, 15) in the RD group with lung involvement, 5.0 pg/ml (IQR 5.0, 5.0) in the RD group without lung involvement and 5.0 pg/ml (IQR 5.0, 5.0) in the control group were the median serum IFN-γ levels. The RD group with lung involvement had substantially higher IFN-γ levels than both the control group and the RD group without lung involvement (*P* < 0.05).

These findings imply that the RD group with lung involvement is characterized by elevated blood levels of IL-6 and IFN-γ.

### 3.4 Diagnostic utility of IL-6 and IFN-γ cytokines concentrations for RD-lung involvement patients

We assessed the diagnostic value of IL-6 and IFN-γ for RD-related lung involvement using ROC curves. AUC (area under the curve) for IL-6 was 0.782 (*P* = 0.005). With a sensitivity of 0.833 and specificity of 0.667, the ideal cut-off value was found to be 6.25 pg/ml. AUC for IFN-γ was 0.742 (*P* = 0.011), and 5.42 pg/ml was determined to be the ideal cut-off value. This threshold produced results with a 0.636 sensitivity and 0.889 specificity (Table 3).

**Table 3:** Predictive performance of IL-6 and IFN-γ based on the ROC analysis.

Variables	Cut-off value (pg/ml)	Sensitivity (%)	Specificity (%)	PPV (%)	NPV (%)	AUC	*P* value
IL-6	6.25	83.3	66.7	62.5	85.7	0.782	0.005*
IFN-γ	5.42	63.6	88.9	77.8	80.0	0.742	0.011*

AUC, area under the curve; PPV, positive predictive value; NPV, negative predictive value.

*P < 0.05 was considered statistically significant.

### 3.5 Correlations between clinical variables and cytokines in RD group with lung involvement

We conducted a Spearman correlation analysis between IL-6, IFN-γ and clinical variables in RD group with lung involvement (Table 4). There was no discernible association between the parameters of PFT and the levels of IL-6 and IFN-γ (*P* *>* 0.05). The IL-6 level and CRP and ESR were shown to be significantly positively correlated. A noteworthy inverse relationship was discovered between IL-6, IFN-γ level and CD4, CD8 (*P* < 0.05). Intriguingly, there was a strong correlation (*P* < 0.001) between the level of IFN-γ and IL-6.

**Table 4:** Spearman correlation between IL-6, IFN-γ levels in RD-lung involvement group.

	Disease duration	CRP	ESR	LDH	CD_4_	CD_8_	FVC%	DLco%	IFN-γ
IL-6	−0.21	0.31*	0.54***	0.23	−0.32*	−0.38**	0	−0.31	0.48***
IFN-γ	−0.17	−0.01	0.18	0.15	−0.36*	−0.33*	0.01	−0.09	NS

CRP, C-reactive protein; ESR, erythrocyte sedimentation rate; LDH, lactate dehydrogenase; FVC, forced vital capacity; DLCO, diffusing capacity of the lung for carbon monoxide; IL-6, interleukin-6; IFN-γ, interferon gamma.

* Indicated that statistically significant *P* < 0.05.

** *P* < 0.01.

*** *P* < 0.001.

## 4 Discussion

This is the first study about cytokine profile in childhood-onset RD patients with lung involvement in China. Newly diagnosed and treatment naive could guarantee eliminating the influence of drugs.

In the study, We found The RD group with lung involvement had considerably greater levels of IL-6 and IFN-γ than the RD group without lung involvement. The measurement of IL-6 and IFN-γ maybe a useful biomarker of lung involvement in RD patients.

Rheumatic disorders often involve activation of auto-inflammatory and autoimmune processes, which can lead to pulmonary dysfunction (19). Even though children's symptoms might not be noticeable at first or not at all, they can lead to significant morbidity and mortality (2). While children generally exhibit fewer pulmonary manifestations than adults, PFT abnormalities have been reported in as many as 48% of a pediatric sample (20). Our current investigation revealed a high prevalence (41.5%) of pulmonary involvement in newly diagnosed RD cases, as assessed through functional or structural assessments. This finding aligns with our previous observations (21). Since the patients are asymptomatic, the prevalence of lung involvement varies depending on the detection method. Advanced screening modalities play a crucial role in early detection and intervention.

While the precise pathogenic mechanisms underlying lung involvement remain unclear, some research suggests that cytokines dysregulation—a crucial component of the immune system—plays a major role in the development of lung involvement (22).

Cytokines, active participants in an intricate network of connections, govern immunoregulatory processes and the inflammatory response. In adult-related research, cytokines have been identified as new biomarkers that might help focus screening efforts on people who are most likely to acquire a disease, advance it, and respond well to therapy (3).

Acute-phase pleiotropic inflammatory cytokine (IL-6) is generated by a variety of cells, including fibroblasts, monocytes, and lymphocytes. It contributes to the acute phase response, T-cell activation, and stimulation of megakaryocytic and myeloid progenitors in the hematopoietic system (23). IL-6 levels were reported to be slightly elevated in a rat model of experimental pulmonary fibrosis, which was linked to a proliferative response in fibroblasts from idiopathic pulmonary fibrosis (IPF) (24). Attenuated bleomycin-induced inflammatory cell buildup and subsequent fibrotic lung alterations were observed in IL-6-deficient mice (25).

Cui et al. identified IL-6 was the primary cause of progressive fibrosis in fibrotic lungs. They demonstrated that fibroblasts expressing *JUN* upregulate IL-6 production and release, which affects the innate and adaptive immune systems (26). Later on in the illness, IL-6 mediates the advancement of fibrosis by activating the IL-6/STAT3 and TGF-β signaling pathways in turn (27).

According to our current investigation, pediatric RD patients with lung involvement had considerably greater amounts of IL-6 in their sera than did those without and healthy controls. This suggests that the elevated level in sera closely corresponds with the emergence of abnormalities in the lung. Higher serum IL-6 levels were helpful in predicting lung involvement, according to ROC curve analysis. According to Lee et al., elevated serum IL-6 levels can independently predict acute exacerbation and serve as a prognostic factor for ILD patients’ death (28). Nara et al's findings suggest that serum IL-6 levels may be a helpful prognostic indicator for clinically amyopathic dermatomyositis with rapidly progressive interstitial lung disease, influencing decisions on the intensity of early-phase immunosuppressive treatment (29).

We discovered that IL-6 cytokine was a statistically significant marker for lung involvement in RD patients, which is consistent with the literature.

According to De Lauretis et al., serum IL-6 was predictive of FVC and DL_CO_ declines within the first year in SSc patients, with a threshold of 7.7 pg/ml (30). But we did not find a relationship between IL-6 and PFT findings.

Additionally, compared to RD patients without lung involvement, our study showed a considerably greater level of IFN-γ in lung involvement RD patients.

Type 1 CD4 and CD8 T cells, as well as natural killer (NK) cells, produce the main effector cytokine, IFN-γ (31). Reports on the clinical efficacy of IFN-γ in lung involvement have been contradictory. IFN-γ has been shown by Ishikawa et al. to have a major role in the pathogenesis of dermatomyositis (DM), particularly in the development of pulmonary lesions that are seen in rapidly progressing interstitial lung disease (RP-ILD) (32). By stimulating macrophages and quickening inflammation, IFN-γ seems to have a pathogenic effect on DM RP-ILD. In Iriguchi S et al's study, they found mice with T-bet overexpression in T lymphocytes, driving IFN-γ production resulting in erythrophagocytosis, as well as alveolar macrophage dysfunction, leading to PAP-like lung disease (33).

Furthermore, IFN-γ can accelerate the development of pulmonary fibrosis via controlling cell apoptosis (34). Prior to Fas activation, exposure to IFN-γ markedly boosted the activity of caspase 3, 7, and 8, as well as the processing of CK-18, a crucial cytoskeletal protein in human lung epithelial cells, by caspase. Additionally, it raised the appearance of apoptotic nuclei (35).

However, the roles of IFN-γ in lung maybe a double-edged sword. According to certain studies, TGF-beta-induced extracellular matrix (ECM) synthesis by myofibroblasts is negatively regulated by IFN-γ, exhibiting anti-fibrotic action (36). IFN-γ prevents fibroblast development into myofibroblasts by opposing TGF-β1 signaling, in part by upregulating SMAD7 and antagonizing SMAD and the JAK pathway at the nuclear p300/CBP level (37). The functions and mechanisms of IFN-γ in lung injury associated with RD are yet to be clarified and need more extensive clinical trials.

We discovered a strong inverse relationship between IL-6, IFN-γ, and CD4, CD8 in our investigation. It's interesting to note that IL-6 and IFN-γ were shown to positively correlate in this investigation. According to a research by Huang et al., blood lymphopenia in anti-MDA5 + patients is probably caused by lymphocytes being transferred to the lungs to take part in the local immune response, which lowers the amount of free lymphocytes in the blood (38). We speculate that the combined upregulation of IL-6 and IFN-γ facilitates lymphocyte egress from the circulatory system into the lungs and thus participates in the process of lung pathology. Further studies are necessary to validate these results.

Finally, it is recommended that patients with recently diagnosed RD who have elevated levels of serum IL-6 or IFN-γ pay great attention to any modest pulmonary involvement. Pulmonary specialists can provide guidance on early detection techniques for impaired pulmonary function.

The study has some limitations. First, a somewhat small population from a single center participated in this retrospective study. Second, the study included patients with different subtypes of diseases. Finally, our study did not include patients who were unable to complete the PFT test, introducing a potential selection bias. Thus, a longer-term study with a more extensive RD population might be beneficial for future investigations. To our knowledge, however, this is the first study examining the relationships between cytokines and pulmonary involvement in Chinese children who have recently been diagnosed with rheumatic disorders but have not yet received therapy.

## 5 Conclusion

A vital component of the immune system, cytokine dysregulation greatly influences the etiology of illness. This study shows that among RD patients with lung involvement, serum levels of IFN-γ and IL-6 were higher. Patients with childhood RD may be at risk for lung illness if they have elevated serum levels of the cytokines IL-6 and IFN-γ.

## Data Availability

The raw data supporting the conclusions of this article will be made available by the authors, without undue reservation.
